# Instructional design complexity and pop-up notification interference: effects on attention allocation and information retention in virtual classrooms

**DOI:** 10.3389/fpsyg.2025.1618121

**Published:** 2025-11-12

**Authors:** Yushan Wang

**Affiliations:** School of Foreign Languages, Huaiyin Normal University, Huai'an, Jiangsu, China

**Keywords:** instructional design complexity, pop-up notification interference, attention allocation, eye tracking, multimedia learning, virtual learning, cognitive load theory, educational technology

## Abstract

The growing prevalence of virtual multimedia learning environments raises questions about how instructional complexity and environmental interference jointly shape learning. This study examines the independent and interactive effects of instructional design complexity (IDC) and pop-up notification interference (PNI) on attention allocation and information retention. IDC was manipulated through instructional design, using streamlined layouts with concise text (low complexity) versus fragmented layouts with redundant on-screen text and background audio (high complexity). PNI (external-to-material) was manipulated through the presence or absence of periodic, task-irrelevant pop-up notifications. Drawing on Cognitive Load Theory (CLT), the Limited Capacity Model of Mediated Message Processing (LC4MP), and Media Multitasking Theory (MMT), a 2 × 2 between-subjects experiment was conducted with 240 Chinese undergraduates. Both IDC and PNI had significant adverse main effects, and their combination produced the lowest attention and retention scores. Structural equation modeling revealed that attention allocation partially mediated the relationship between the two factors and retention performance. Moderation analysis showed that learners with greater digital learning experience were less affected by PNI. This research advances CLT in ecologically valid digital contexts. It offers actionable design principles for creating distraction-resilient, cognitively sustainable virtual learning environments by integrating process-level attention metrics with clearly defined dual-factor manipulations.

## 1 Introduction

The exponential rise of virtual learning platforms has transformed how students engage with instructional materials, creating new demands on cognitive processing in multimedia environments (Clark and Mayer, 2023). This shift has increased the use of multimedia designs that integrate text, images, video, animation, and interactive elements (Clark and Mayer, 2023; Noetel et al., 2022). While these features can enrich learning experiences, they may also strain limited working memory capacity. Within this evolving landscape, CLT provides a framework for understanding how the structure and presentation of information influence learning outcomes by balancing intrinsic, extraneous, and germane cognitive loads (Skulmowski and Xu, 2022). Specifically, learners face internal-to-material features, most commonly fragmentation/split-attention layouts and redundancy, as well as embedded background audio, all of which are linked to increased processing demands and poorer learning (Schroeder and Cenkci, 2018; Trypke et al., 2023; Souza and Barbosa, 2023; de la Mora Velasco et al., 2023). In parallel, external-to-material events such as mobile-style pop-up notifications and other on-screen interruptions reliably capture attention and impair task performance (Stothart et al., 2015; Ohly and Bastin, 2023; Castelo et al., 2025), consistent with contemporary accounts of stimulus-driven attentional capture (e.g., Gaspelin and Luck, 2018). Accordingly, we adopt operational labels (IDC internal; PNI external) and refrain from attributing unique mechanisms, in line with the construct validity of cause logic (Shadish et al., 2002). Empirically, fragmentation and redundancy increase processing demands and depress learning, i.e., the split-attention/contiguity and redundancy families document this consistently (Ginns, 2006; Adesope and Nesbit, 2012; Trypke et al., 2023), and abrupt visual onsets (the perceptual class to which pop-ups belong) capture attention exogenously and disrupt goal-directed processing (Zhang et al., 2025).

Despite extensive literature examining design-induced demands or notification-driven interference individually (e.g., Chen and Yan, 2016; May and Elder, 2018), there is limited knowledge of their interactive effects in authentic virtual learning contexts (Liao and Wu, 2022; Mark, 2022). In such contexts, learners often face dual cognitive demands, high informational complexity, combined with persistent environmental interference, which are rarely studied together (Sharma and Saxena, 2025). What is missing is a rigorous investigation that disentangles these constructs conceptually and methodologically, and tests how they jointly shape attention mechanisms (allocation, shifts, and maintenance) and learning outcomes (retention and application).

Guided by CLT (Sweller, 1988), the LC4MP (Rössler, 2017), and MMT (Zamanzadeh and Rice, 2021), this study adopts a process-oriented perspective. CLT explains how poorly designed materials increase extraneous cognitive load (ECL), reducing available capacity for schema acquisition. LC4MP describes how finite cognitive resources are dynamically allocated during media processing, with greater message complexity and competing stimuli taxing both automatic and controlled attentional processes. The MMT, supported by meta-analytic evidence on multitasking costs (Parry and Le Roux, 2021), posits that habitual multitasking can weaken executive control and increase switching costs. In this study, media multitasking refers specifically to the management of multiple streams of media-based information, of which digital distraction is a particular case.

The unique contribution of this work lies in its dual-factor experimental design, i.e., a controlled 2 × 2 manipulation of IDC and PNI, combined with real-time attention measures (time-on-task, response latency, eye-tracking fixation data) to examine both independent and interactive effects. The study moves beyond the simple logic of a manipulation check by clearly separating instructional features from environmental interference and grounding the manipulations in established theory. It provides evidence on how instructional complexity and distraction jointly influence attention and retention, offering an essential step toward designing virtual learning environments (VLEs) that are both cognitively sustainable and resilient to distraction.

## 2 Literature review

### 2.1 Cognitive load theory and multimedia learning

The CLT provides a cognitive account of how instructional design modulates learning by taxing limited working memory resources. Contemporary measurement syntheses converge on three load types (intrinsic, extraneous, and germane) while also noting empirical entanglements among them in practice (Krieglstein et al., 2022). In the present work, the term ‘extraneous load’ is used strictly as a theoretical construct. At the operational level, we manipulate IDC (e.g., split-attention, redundancy), which aligns with recent recommendations for digital learning to separate design-induced demands from concurrent environmental events, avoiding the conflation of construct and manipulation (Skulmowski and Xu, 2022). From a learner’s perspective, internal features are integrated while alerts are suppressed (see 2.5 for the processing-level prediction and 3.3.2 for operational indices).

In this study, IDC targets established design pitfalls (namely spatial split-attention/fragmentation and content or modal redundancy), which elevate processing demands and, under common conditions, hinder learning in multimedia materials (Schroeder and Cenkci, 2018; Trypke et al., 2023; Albers et al., 2023). Evidence syntheses also describe when dual-modality (e.g., narrated animation) reduces processing demands (the modality effect) relative to text-heavy visuals (Castro-Alonso et al., 2021), while adding written text to narrated or pictorial streams often harms learning unless tightly integrated (Leahy and Sweller, 2011; Trypke et al., 2023; Albers et al., 2023). Translating these regularities to notification-rich delivery requires recognizing that environmental events can simultaneously compete for limited processing resources as formalized by resource-allocation models (LC4MP).

### 2.2 Attention allocation in learning

Attention gates encode, store, and retrieve information. In mediated environments, the LC4MP frames learning as a competition for a finite pool of processing resources among encoding, storage, and retrieval subprocesses (Fisher et al., 2018a). Process-level measures now quantify attention with increasing precision. Recent studies have linked fixations, dwell time, and gaze synchrony to selection/integration processes and to learning indicators in video-based instruction (Deng and Gao, 2023; Bühler et al., 2024). Despite methodological advances, attention is still infrequently modeled as a mediator in experiments combining design manipulations with environmental events. Exceptions demonstrate tractability. In a semester-long online course, augmenting videos with the instructor’s gaze improved conceptual learning, with joint visual attention mediating the effect (Schneider and Sung, 2024). Complementarily, a contemporary cognitive-process framework for digital learning emphasizes specifying mediating mechanisms to enable causal explanation using digital traces (Reinhold et al., 2024).

From the learner’s perspective, internal features of the material (IDC) are tagged as to-be-learned and therefore become part of the task set, whereas pop-up alerts (PNI) are tagged as task-irrelevant. This difference predicts distinct control policies, i.e., either integrating IDC elements or suppressing PNI. Two attention traditions support this claim. First, contingent capture, where capture depends on current goals/task set (Folk et al., 1994; Remington et al., 2001), and second, signal suppression, where salient but irrelevant onsets are proactively inhibited (Gaspelin et al., 2025). Notifications still impose costs even when ignored (Stothart et al., 2015), but learners can and do attempt to suppress them. Conversely, cues that are internal-to-material guide attention and perceived relevance (signaling principle), improving selection/integration (Schneider et al., 2018; Lai and Zhang, 2021).

### 2.3 Digital distraction and media multitasking

Task-irrelevant PNI is a pervasive barrier to focused learning online. Brief alerts capture attention and impair performance, even without interaction, in controlled and field settings (Stothart et al., 2015; Castelo et al., 2025). Mechanistically, contemporary accounts of attention explain capture via stimulus-driven priority signals and the need for active suppression (Gaspelin and Luck, 2018). Media multitasking (MMT) is a trait-like tendency to engage with multiple streams concurrently (Ophir et al., 2009). Meta-analytic and cumulative evidence links heavier MMT to small but reliable decrements in executive control (Wiradhany and Nieuwenstein, 2017; Parry and Le Roux, 2021) and to negative effects on learning in instructional tasks (Jeong and Hwang, 2016; Haverkamp et al., 2024). In educational contexts, heavier media multitasking is associated with poorer note-taking, reduced comprehension, and lower test performance (Haverkamp et al., 2024). Importantly, situational PNI impairs attention and memory independently of habitual multitasking status, including in relatively simple tasks (Stothart et al., 2015). These constructs converge on the prediction that frequent attentional switching (trait-like or situational) undermines control processes and reduces encoding efficiency.

### 2.4 Integrating cognitive load and distraction in virtual learning

While CLT and MMT literatures have progressed in parallel, integration remains limited. For digital learning, recent CLT work explicitly urges disentangling design-induced demands from interruptions to avoid construct confounds (Skulmowski and Xu, 2022). Contemporary distraction syntheses, in turn, foreground resource competition and interruption costs across modalities (Marsh et al., 2024). Empirical tests of attentional mediation under concurrent demands are relatively sparse, as most studies prioritize global outcomes. Fine-grained attention measures (e.g., fixations, dwell time, gaze synchrony) remain underutilized in interaction tests, despite their links to selection/integration processes (Deng and Gao, 2023; Bühler et al., 2024). The present design treats IDC (internal, design-level) and PNI (external, task-irrelevant alerts) as theoretically orthogonal yet jointly resource-competitive. LC4MP predicts competition for encoding/storage resources under concurrent stimuli (Fisher et al., 2018b), and process frameworks emphasize specifying mediators to enable causal explanation with digital traces (Reinhold et al., 2024).

### 2.5 Conceptual model and research hypotheses

**Figure 1:**
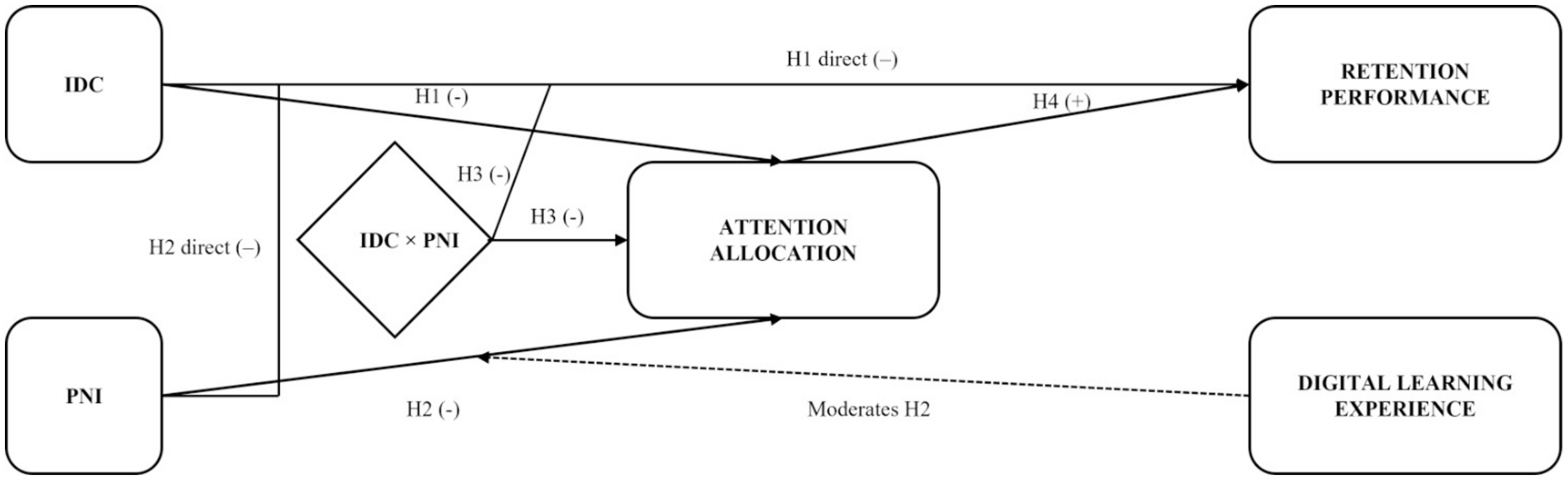
Conceptual model. Solid arrows denote main and interaction effects; dotted arrow denotes the moderation of the PNI and attention path by Digital Learning Experience.

Understanding how learners process and retain information in digital multimedia environments requires a clear distinction between instructional features, cognitive processes, attentional mechanisms, and learning outcomes. The conceptual framework guiding this study is illustrated in Figure 1, which depicts the hypothesized relationships among the core variables: IDC, PNI, attention allocation, and retention performance. In this model, IDC (internal-to-material) and PNI (external-to-material) are treated as independent variables. IDC is appraised as to-be-learned and integrated, whereas PNI is appraised as irrelevant and actively suppressed (contingent capture/signal suppression), predicting distinct control policies, which we later index with eye-movement measures (see 3.3.2). Attention allocation, operationalized as total dwell time (seconds) within predefined core AOIs (see 3.3.2 for operational indices; proportional robustness analyses are reported in Appendix A), functions as the mediating mechanism through which these factors influence learning outcomes, measured by immediate and delayed retention performance. Digital learning experience is positioned as a moderator, hypothesized to buffer the negative effect of distraction on attention allocation. Solid arrows represent hypothesized direct and mediated relationships, while the dotted arrow represents the moderation pathway. This model integrates predictions from three complementary cognitive frameworks into a unified structure for analyzing cognitive performance in virtual multimedia learning environments.

The CLT predicts deteriorating learning when non-essential processing consumes limited capacity. At the operational level, split attention and redundancy are canonical IDC sources that draw resources without commensurate learning benefits. Recent meta-analyses and reviews have shown that minimizing these elements improves outcomes across various contexts (Schroeder and Cenkci, 2018; Çeken and Taşkın, 2022; Trypke et al., 2023; Albers et al., 2023). Consistent with CLT and the limited-capacity model of motivated, mediated message processing, we treat attention allocation as the learner’s control policy for prioritizing goal-relevant instructional content over peripheral or task-irrelevant events. In practice, increases in IDC demand more selection and integration across elements, whereas PNI produces exogenous capture that must be actively suppressed. Accordingly, our model predicts that attention (indexed by dwell time on core Areas of Interest (AOIs), with complementary indices detailed in §3.3.2) will decline as either IDC or PNI increases, and that lower attention will, in turn, impair retention; hence,

*H1*: High instructional design complexity will reduce attention allocation to core content and lower retention relative to low complexity.

This learner-appraisal logic yields a prediction at the processing level. IDC elevates integration demands (longer, redistributed fixations), whereas PNI elicits suppress-or-switch responses with brief, attention-grabbing onsets. Both routes can degrade encoding, albeit via different control policies (Gaspelin et al., 2025; Stothart et al., 2015).

Moreover, contemporary accounts of attention treat it as a limited cognitive resource that must be strategically allocated across encoding, storage, and retrieval (Wickens, 2020). In digital settings, notification-driven interruptions tax this capacity, redirecting resources away from learning-relevant material. Thus:

*H2*: High PNI will reduce attention allocation to core content and lower retention relative to no/low notifications.

In authentic digital learning, IDC and PNI often co-occur. CLT implies that when IDC is high, non-essential processing consumes resources; LC4MP further predicts simultaneous competition for encoding/storage when external stimuli intrude (Fisher et al., 2018b). Recent distraction syntheses similarly highlight the costs of interruption and cumulative strain from concurrent demands (Marsh et al., 2024). Hence:

*H3*: The combination of high instructional design complexity and high pop-up notification interference will produce a greater negative effect on attention and retention than either factor alone.

Within CLT and LC4MP, attention is the bottleneck that determines how external conditions translate into learning. Here, attention allocation is defined as visual/cognitive focus to relevant AOIs (fixation proportion, time-on-task). Empirically, attention can mediate design effects. In a semester-long online course, joint visual attention mediated the benefit of gaze-augmented videos for conceptual learning (Schneider and Sung, 2024). Process frameworks likewise urge explicit mediator specification with digital traces (Reinhold et al., 2024). Therefore:

*H4*: Attention allocation to relevant instructional content will partially mediate the effects of instructional design complexity and pop-up notification on retention.

*H4a*: Higher instructional design complexity will reduce attention to relevant content, leading to lower retention.

*H4b*: Greater pop-up notification will reduce attention to relevant content, leading to lower retention.

## 3 Methodology

### 3.1 Research design

The study was conducted in a controlled VLE built on a custom web-based multimedia platform developed using HTML5 and JavaScript. The platform was optimized for desktop use, enabling precise integration of multimedia elements and embedded experimental controls. The learning module, titled “Climate Change and Carbon Neutrality,” comprised 18 instructional slides that incorporated narrated animations, explanatory diagrams, infographics, and strategically placed visual cues. The low-complexity version adhered to multimedia design principles, whereas the high-complexity version implemented IDC (internal-to-material) via spatial fragmentation/split-attention layouts, redundant on-screen text, and background audio. Split-attention/contiguity and redundancy reliably increase processing demands and impair learning, and lyrics in music often degrade cognitive performance in learning tasks (Schroeder and Cenkci, 2018; Trypke et al., 2023; Souza and Barbosa, 2023). In high-PNI conditions, 8 visual pop-ups (size 360 × 120 px) appeared in the top-right quadrant for 1.5 s each (no sound), with onsets pseudo-randomly jittered (45–105 s; mean ≈75 s) to prevent anticipation. Low-PNI conditions presented 0 pop-ups. Pop-up content was neutral (generic system notifications) and unrelated to lesson content. Placement avoided occluding core AOIs. These manipulations were pre-validated in a pilot (*n* = 32) using the 9-point Paas Mental Effort scale and a brief comprehension test (Paas, 1992; Ouwehand et al., 2021). To avoid measurement reactivity and because single-item loadings show mixed validity, we did not administer in-session ECL questionnaires (see 5.4 for construct-level implications and future measurement plans). Each slide was presented in a fixed sequence, with embedded comprehension prompts, and the session lasted 20 min. No VR elements were included, as all interactions took place within the 2D interface of the VLE to ensure experimental control and compatibility with the eye-tracking system. Eye movements were recorded with Tobii Pro Fusion remote eye-trackers (250 Hz), a screen-based system suitable for controlled 2D interfaces.

Exemplar screenshots of the learning materials for each condition are provided with AOIs overlaid. Appendix Figure A1 (Low IDC + Low PNI) shows a clean layout with a single content panel (AOI-Core). Appendix Figure A2 (High IDC + Low PNI) adds redundant on-screen text (AOI-Peripheral) and split-attention elements (shaded bands) to illustrate fragmentation. Appendix Figure A3 (Low IDC + High PNI) overlays a task-irrelevant pop-up (AOI-Popup) on the clean layout. Appendix Figure A4 (High IDC + High PNI) combines fragmentation/redundancy with a concurrent pop-up. AOIs were defined *a priori* to support process-level inferences, i.e., AOI-Core (essential text/diagram), AOI-Peripheral (redundant/verbal overlays), and AOI-Popup (task-irrelevant notification). These AOIs map directly onto our measures of attention allocation (fixation duration, dwell time within AOIs) and event-based transition analyses (e.g., Popup → Core, Core → Peripheral), in line with minimal reporting standards for eye-tracking and recent guidance linking constructs to eye-movement proxies (Hessels et al., 2024).

### 3.2 Participants

A total of 240 undergraduate students (aged 18–25) from three universities in Beijing participated in the study. Recruitment was conducted through departmental bulletin boards, class WeChat groups, and university-wide email lists. Participants were compensated with either a ¥70 honorarium (~USD 10) or equivalent course credit upon completion. Stratified sampling ensured balanced representation across gender and academic disciplines, maximizing demographic and cognitive diversity. Before random assignment, all volunteers completed a 5-item prior knowledge test on climate change and carbon neutrality. Participants scoring more than ±1 SD from the mean were excluded to reduce ceiling and floor effects. Eighteen individuals met this exclusion criterion and were replaced to maintain equal group sizes. This process resulted in four experimental groups (Low IDC–Low PNI, Low IDC–High PNI, High IDC–Low PNI, High IDC–High PNI), each with 60 participants. An a priori power analysis targeted a medium effect (*η*^2^ ≈ 0.06; *f* ≈ 0.25) at *α* = 0.05, 1–*β* = 0.80. Benchmarks for design-induced demands are consistent with medium effects for integrated vs. split-attention layouts (g ≈ 0.63) and small-to-moderate performance costs from notifications/interruptions, supporting a medium assumption for main/interaction tests (Schroeder and Cenkci, 2018; Stothart et al., 2015). This informed the a priori power analysis parameters, ensuring adequate sensitivity to detect both main and interaction effects.

### 3.3 Independent variables and experimental manipulations

#### 3.3.1 Two independent variables were manipulated: IDC and PNI

This study manipulated two independent variables at the operational level. IDC within the learning materials and PNI within the environment. IDC combined visual (fragmentation/split-attention, redundancy) and auditory (background audio) elements typical of multi-element multimedia lessons. These features reliably elevate processing demands and can impair learning (Schroeder and Cenkci, 2018; Trypke et al., 2023; Souza and Barbosa, 2023). Because PNI was implemented visually (with abrupt on-screen onsets), whereas IDC bundled visual and auditory features, the design does not isolate within-visual IDC from auditory-only elements. Consequently, any IDC × PNI interaction may be partly attributable to matched-modality competition in the visual channel, as predicted by multiple resource theory and recent interruption work (Nason and Wilbiks, 2025; Wickens, 2024). PNI consisted of abrupt-onset, task-irrelevant pop-ups. Abrupt onsets capture attention exogenously and degrade performance even without user interaction (Zhang et al., 2025). Accordingly, any H3 interaction would be interpreted as compounding demands under the present modality configuration, rather than a general cross-modal interaction. We interpret the results at the operational level (IDC, PNI) rather than as unique signatures of a single load type, and refrain from construct-level claims about ECL without direct measurement.

Measures included a 5-item prior knowledge test (multiple-choice) administered before random assignment, with only participants scoring within ±1 standard deviation of the mean retained. Example item: “*What is the primary greenhouse gas responsible for global warming*?” Retention was assessed using immediate and delayed post-tests, each containing 10 multiple-choice and 5 short-answer items that targeted comprehension and application levels of Bloom’s taxonomy. Example multiple-choice item: “*Which strategy most effectively reduces carbon emissions*?” Example short-answer item: “*Explain the relationship between carbon neutrality and renewable energy adoption*.” The delayed test was administered 48 h later to assess short-term retention. Retrieval-practice syntheses demonstrate that the advantages of learning manipulations typically emerge or stabilize at delays of days (McDermott, 2021; Roediger and Karpicke, 2006). Additional measures included the Digital Learning Experience Scale (7-point Likert) with items such as “I feel confident navigating different types of online learning platforms,” and the Self-Reported Distraction Susceptibility Scale (7-point Likert) with items such as “*I often check my phone during online lectures*.”

#### 3.3.2 Dependent variables

The study measured two dependent variables, i.e., attention allocation and information retention. Attention allocation was defined as total dwell time (seconds) in core instructional AOIs, computed from fixation-level gaze data within predefined AOIs containing essential text and visuals (Ni et al., 2025; Hübner et al., 2025). Event-locked pop-up indices are treated as primarily indexing bottom-up capture (shorter time-to-first-fixation (TTFF) and greater 0–2000 ms pop-up dwell reflect exogenous, onset-driven capture), whereas pass-based core-AOI measures index top-down integration and repair (longer first-pass gaze reflects goal-directed integration. Greater second-pass time and higher regression-back probability reflect controlled re-orientation). Transition probabilities (pop-up → core within 1,000 ms; baseline core → pop-up) quantify recovery from capture (top-down suppression) versus susceptibility to upcoming onsets. This mapping follows contingent-capture and signal-suppression accounts, as well as standard interpretations from eye-movement research (Folk et al., 1992; Rayner, 2009; Gaspelin and Luck, 2018).

Eye movements were recorded using Tobii Pro Fusion remote eye-trackers (250 Hz), capturing fixation duration, fixation count, and saccades within AOIs. Beyond standard fixation duration, count, and dwell time, we analyzed process-sensitive indicators recommended in recent eye-tracking guidance (Liu and Cui, 2025; Hessels et al., 2024) and foundational reading research (Rayner, 2009). To capture immediate attentional capture by notifications, we computed event-locked pop-up measures: TTFF in the pop-up AOI after onset (ms) and pop-up dwell time within the 0–2000 ms window following onset. To characterize shifts of attention between regions, we estimated transition probabilities from the pop-up AOI to the core-content AOI within 1,000 ms of the first pop-up fixation, as well as the reverse transition (core → pop-up) during a −2000 to 0 ms baseline window before onset, quantifying capture and recovery. We also distinguished early versus later processing of core AOIs by measuring first-pass gaze duration (the sum of fixations before gaze first exits an AOI), rereading or second-pass time (the sum of fixations after an AOI is revisited), and regression-back probability as the likelihood of returning to a previously viewed core AOI. We interpret longer first-pass durations as increased initial integration demands and greater second-pass time/regressions as repair or reintegration effort (Rayner, 2009).

Complementary behavioral engagement metrics, including response latency, time-on-task, and task-switching frequency, were extracted from VLE logs. Information retention was measured through two assessments. An immediate post-test was administered directly after the session, and a delayed post-test was administered 48 h later. Each test consisted of 10 multiple-choice and 5 short-answer items, developed in collaboration with subject-matter experts, to assess retained comprehension and application of the learned material. Example items included: “*Which of the following gasses has the highest global warming potential over a 100-year period*?” and “*Explain how carbon neutrality can be achieved at a national level, citing two key strategies*” (short-answer). To justify using a single retention score, we examined the psychometrics of the comprehension (MCQs) and application subscales. Internal consistencies were *α*/ω_comprehension = 0.82/0.84 and α/ω_application = 0.79/0.81. The subscales correlated *r* = 0.66, 95% CI [0.58, 0.73] (*N* = 240). We then compared a one-factor confirmatory model with a two-factor confirmatory model on the immediate test. The one-factor solution yielded CFI = 0.957, RMSEA = 0.055, and SRMR = 0.046, whereas the two-factor solution resulted in CFI = 0.964, RMSEA = 0.049, and SRMR = 0.040. Given the substantial correlation and acceptable unidimensional fit (with a modest ΔCFI = 0.007), we proceeded with a composite retention score for the primary analyses (common reporting heuristics: CFI ≈ 0.95, RMSEA≈0.06, SRMR≈0.08).

#### 3.3.3 Moderation analysis

Digital learning experience was assessed using a self-report scale adapted from the Digital Learning and Multitasking Inventory. Participants rated their familiarity and comfort with online learning tools, including learning management systems, video conferencing platforms, and interactive multimedia modules, as well as the frequency of their engagement in virtual courses over the past 12 months. Sample items included: “*How confident are you in navigating a learning management system (e.g., Moodle, Blackboard)*?” and “*How often have you participated in interactive multimedia-based lessons in the past year*?” Responses were recorded on a 7-point Likert scale (1 = very low, 7 = very high) and averaged to yield a composite score; higher values indicate a greater digital learning experience. The scale demonstrated strong internal consistency in this study (Cronbach’s *α* = 0.88).

#### 3.3.4 Controlled variables

Multiple control variables were implemented to ensure the integrity of causal inference. First, prior knowledge on the learning topic was measured through a 5-item pre-test. Only participants whose scores fell within ±1 standard deviation of the sample mean were retained to eliminate ceiling and floor effects. Language proficiency was controlled by limiting participation to native Mandarin speakers, and all instructional materials were delivered in simplified Chinese. Environmental factors were also standardized. All participants used identical laptops (1920 × 1,080, 60 Hz) with standardized audio output. Sessions were conducted in a dedicated computer lab with controlled lighting and sound insulation. The testing sessions were held between 10:00 a.m. and 1:00 p.m. to minimize circadian variations in attention and alertness. Participants were also instructed to refrain from using personal devices during the task.

### 3.4 Procedure

Participants were randomly assigned to one of the four experimental conditions upon arrival. After completing informed consent and the pre-test, they underwent a five-point calibration procedure for the eye-tracking system. Participants were given a brief orientation to the virtual learning platform and instructed not to navigate away from the screen during the session. They then completed a 20-min multimedia lesson aligned with their assigned condition (e.g., high IDC + high PNI). During this phase, eye movements and behavioral interaction data were recorded continuously. Immediately after the learning session, participants completed the post-test. The delayed retention test was administered online 48 h later, with reminders sent via the university’s email system. The 48-h delay was chosen to capture short-term retention decay while minimizing potential interference from unrelated academic activities. Throughout the experiment, trained research assistants closely monitored participants to ensure protocol compliance and addressed technical issues in real time.

Multiple-choice items were automatically scored by the VLE to eliminate scorer bias. Short-answer responses were evaluated using a rubric-based scoring system by two independent raters who were blind to participants’ condition assignments. Inter-rater reliability was high (Cohen’s *κ* = 0.85), and any scoring discrepancies were resolved through discussion, ensuring both objectivity and consistency in the assessment process.

### 3.5 Data analysis plan

Data were analyzed using a combination of SPSS AMOS 29, R (lavaan package), and Tobii Pro Lab software. To assess the primary hypotheses, a series of 2 × 2 between-subjects ANOVAs was used to evaluate the main and interaction effects of IDC and PNI on attention and retention scores. Mixed ANOVAs were used to compare immediate and delayed retention across conditions, allowing for the assessment of memory decay over time. Mediation hypotheses were tested using bootstrapped structural equation modeling (SEM) with 5,000 resamples to generate confidence intervals for indirect effects. The path model included IDC and PNI as exogenous variables, attention allocation as the mediator, and retention performance as the outcome. Model fit indices, such as RMSEA, CFI, and SRMR, were reported to evaluate the model’s adequacy. For the 2 × 2 ANOVAs, we conducted simple effects tests and planned contrasts (each cell vs. the Low IDC–Low PNI control), and we report estimated differences with 95% CIs and standardized effect sizes. This approach adheres to best practices for interpreting interactions and avoids dichotomous ‘buffering/mitigation’ claims (Lakens, 2013; Hayes, 2022; Preacher et al., 2006; Spiller et al., 2013).

For eye-tracking, we exported AOI-level time series and event logs. Beyond heatmaps and scanpaths, we conducted two complementary analyses. Event-locked windows centered on pop-up onset (−2000 to +2000 ms) to estimate TTFF and 0–2000 ms dwell on the pop-up AOI, as well as transition probabilities between regions (pop-up → core and core → pop-up). In addition, first-versus second-pass processing on core AOIs to obtain first-pass gaze duration, second-pass (rereading) time, and regression-back probability. Statistical inference proceeded with 2 × 2 between-subjects ANOVAs (IDC × PNI) for each metric, supplemented for the event-locked windows by linear mixed-effects models with participants as random intercepts and IDC, PNI, and their interaction as fixed effects. TTFF was log-transformed where appropriate, and all results are reported with effect sizes and confidence intervals in line with eye-tracking reporting guidance (Hessels et al., 2024). Full numeric outputs (means, SEs, CIs, and model coefficients) are provided in Appendix Tables A1–A3. Fixations were identified using a velocity-threshold algorithm (I-VT; velocity cutoff: 30°/s; minimum fixation duration: 60 ms). Samples with a tracking ratio < 80% on a slide were excluded listwise for that slide; blinks and track losses were interpolated if gaps < 75 ms. AOIs were pre-registered masks; to reduce boundary artifacts, we applied a 3 px inflation during export. All analyses were conducted on screen-mapped coordinates at 1920 × 1,080 with a sampling rate of 250 Hz.

## 4 Results

### 4.1 Descriptive statistics and assumption checks

Descriptive statistics for attention allocation (measured in seconds) and retention scores (measured as percentage correct) across the four experimental conditions are presented in Table 1. The table also reports the number of participants contributing to each analysis, with the final two columns clarifying sample sizes for attention and retention data separately. Visual inspection of histograms and Q–Q plots, along with Shapiro–Wilk tests, confirmed that residuals met normality assumptions (all *p* > 0.05). Levene’s tests indicated homogeneity of variance for all dependent measures (all *p* > 0.05), supporting the validity of parametric comparisons. Mahalanobis distance screening at *α* = 0.001 detected no multivariate outliers, ensuring that results were not disproportionately influenced by extreme values. Usable eye-tracking data were obtained from 232 participants, while all 240 participants completed the immediate retention test, and 226 completed the delayed test. These sample sizes provided high statistical power for detecting medium effect sizes, and all figures display error bars representing ±1 standard error to aid interpretation of group differences.

**Table 1:** Descriptive statistics.

Condition	Attention allocation (M, sec)	Attention allocation (SD, sec)	Immediate retention (M, %)	Immediate retention (SD, %)	Delayed retention (M, %)	Delayed retention (SD, %)	*n* (attention)	*n* (immediate retention)	*n* (delayed retention)
Low IDC + Low PNI	204.6	14.7	77.2	6.8	71.3	6.9	58	60	57
Low IDC + High PNI	188.1	16.4	71.4	7.2	66.8	7.5	58	60	56
High IDC + Low PNI	187.3	15.2	70.1	8.1	65.2	7.8	58	60	56
High IDC + High PNI	181.2	14.9	62.3	7.8	51.9	6.6	58	60	57

Immediate retention *n* per cell = 60 (total 240). Delayed retention n per cell = 56–57 (total 226).

### 4.2 Main effects of IDC and PNI

Both IDC and PNI exerted significant and practically meaningful negative effects on attention allocation and immediate retention (Table 2). For attention, the effects of IDC (partial *η*^2^ = 0.10) and PNI (partial *η*^2^ = 0.09) were in the medium-to-large range by common benchmarks for partial *η*^2^, with high IDC reducing sustained visual engagement within AOI by an average of 17.3 s compared to low IDC, and high PNI shortening attention by 15.7 s relative to low PNI. For immediate retention, the effects of IDC (partial *η*^2^ = 0.08) and PNI (partial *η*^2^ = 0.07) fell in the medium-to-large range, with performance dropping by an average of 11.8 percentage points under both high IDC and high PNI conditions. According to common reporting guidelines, partial *η*^2^ ≈ values of 0.07–0.10 are typically interpreted as medium to large (Lakens, 2013). The modest interaction (partial *η*^2^ ≈ 0.03) indicates an ordinal (non-crossover) pattern; interpretation is detailed in 4.3.

**Table 2:** Main effects of IDC and PNI on attention allocation and immediate retention.

Dependent variable	Factor	*F*-value	*p*-value	Partial *η*^2^	95% CI (partial *η*^2^)	Mean (low condition)	SD (low condition)	Mean (high condition)	SD (high condition)
Attention allocation	IDC	24.35	<0.001	0.1	[0.04, 0.15]	204.6	14.7	187.3	15.2
Attention allocation	PNI	21.08	<0.001	0.09	[0.03, 0.14]	203.8	13.9	188.1	16.4
Immediate retention	IDC	19.41	<0.001	0.08	[0.03, 0.13]	75.2	7.5	63.4	8.2
Immediate retention	PNI	16.62	<0.001	0.07	[0.02, 0.11]	75.2	7.5	63.4	8.2

### 4.3 Interaction effects

**Figure 2:**
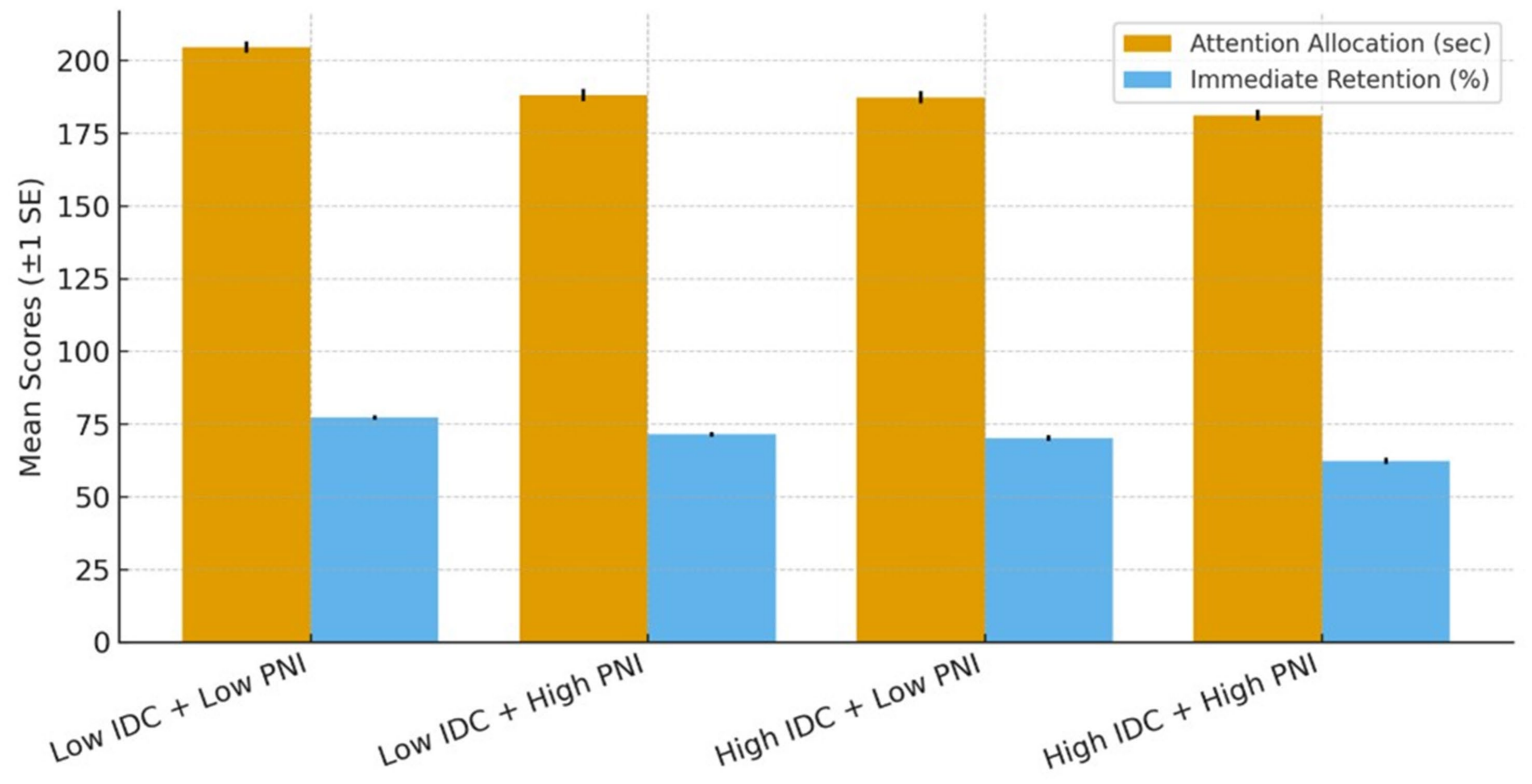
Ordinal (non-crossover) interaction of IDC and PNI on attention and retention.

A significant interaction between IDC and PNI was found for attention allocation, *F*(1, 228) = 6.91, *p* = 0.009, partial *η*^2^ = 0.03, 95% CI [0.005, 0.07], indicating that the effect of one factor depended on the level of the other. Specifically, the high IDC + high PNI group (*M* = 181.2 s, SD = 14.9) showed significantly lower attentional engagement than all other groups (*p* < 0.01, Tukey’s HSD). The pattern was an ordinal (non-crossover) interaction. Low PNI attenuated (but did not eliminate) the negative impact of high IDC, and low IDC attenuated (but did not eliminate) the negative impact of high PNI. Critically, neither attenuated condition returned to the Low IDC–Low PNI control level. For attention, High IDC–Low PNI remained 17.3 s below control (187.3 vs. 204.6), and Low IDC–High PNI remained 16.5 s below control (188.1 vs. 204.6). For immediate retention, High IDC–Low PNI was 7.1 percentage points below control (70.1% vs. 77.2%), and Low IDC–High PNI was 5.8 percentage points below control (71.4% vs. 77.2%). A similar interaction emerged for retention, *F*(1, 236) = 7.42, *p* = 0.007, partial *η*^2^ = 0.03, 95% CI [0.004, 0.06]. To probe the interaction, we tested simple effects and planned contrasts with CIs and effect sizes. Interpretation follows the modality caveat specified in 3.3. These results indicate sub-additive attenuation effects rather than full mitigation (Figure 2).

### 4.4 Delayed retention and memory decay

A 2 (IDC) × 2 (PNI) × 2 (Time: Immediate vs. Delayed) mixed ANOVA revealed a significant main effect of time, *F*(1, 222) = 41.56, *p* < 0.001, partial *η*^2^ = 0.16, confirming that retention scores declined over time across all conditions, indicating overall memory decay. Time also interacted with IDC, *F*(1, 222) = 5.87, *p* = 0.017, partial *η*^2^ = 0.03, and with PNI, *F*(1, 222) = 4.41, *p* = 0.037, partial *η*^2^ = 0.02, suggesting that forgetting rate varied across these factors. The largest decline occurred in the high IDC + high PNI condition (Δ*M* = −10.4%, SD = 3.8, Cohen’s *d* ≈ 1.25), whereas all other conditions showed smaller decreases ranging from −4.6% to −5.9% (Cohen’s *d* ≈ 0.60–0.75). This pattern indicates that the joint presence of high IDC and frequent PNI exacerbated short-term forgetting more than either factor alone. However, the interaction effect sizes (partial *η*^2^ = 0.02–0.03) suggest this amplification, while meaningful, was moderate in magnitude (Table 3).

**Table 3:** Delayed retention and memory decay across experimental conditions.

Condition	Immediate retention (M)	Immediate retention (SD)	Delayed retention (M)	Delayed retention (SD)	Retention decline (ΔM)	SD (ΔM)
Low IDC + Low PNI	77.2	6.8	71.3	6.9	−5.9	3.1
Low IDC + High PNI	71.4	7.2	66.8	7.5	−4.6	3.5
High IDC + Low PNI	70.1	8.1	65.2	7.8	−4.9	3.6
High IDC + High PNI	62.3	7.8	51.9	6.6	−10.4	3.8

### 4.5 Mediation analysis: attention allocation as a mediator

Using bootstrapped SEM with 5,000 resamples, attention allocation was examined as a mediator between IDC, PNI, and retention performance. The model showed good fit (RMSEA = 0.042, CFI = 0.963, SRMR = 0.029), suggesting the hypothesized relationships aligned well with the observed data. Significant but modest indirect effects were found for both predictors. Higher IDC was associated with reduced retention through decreased attention allocation (unstandardized indirect effect *b* = −2.14 percentage points, 95% CI [−3.21, −1.08], small-to-moderate magnitude), and greater PNI showed a similar pathway (unstandardized indirect effect *b* = −1.78 percentage points, 95% CI [−2.85, −0.97], small magnitude). In both cases, direct effects on retention remained significant, indicating partial rather than full mediation. Indirect effects were estimated with a nonparametric bootstrap (5,000 resamples), a recommended practice for mediation inference (Hayes, 2022). These results support H4a and H4b, with the caveat that while the mediation effects are statistically reliable, their magnitudes suggest that other factors beyond attention allocation also contribute meaningfully to the observed performance differences (Table 4).

**Table 4:** Mediation analysis: attention allocation as a mediator between IDC, PNI, and retention.

Pathway	Indirect effect (b, unstandardized, percentage points)	95% CI (indirect effect)	Direct effect significance	Mediation type	Model fit indices
IDC → Attention → Retention	−2.14	[−3.21, −1.08]	Significant	Partial Mediation	RMSEA = 0.042, CFI = 0.963, SRMR = 0.029
PNI → Attention → Retention	−1.78	[−2.85, −0.97]	Significant	Partial Mediation	RMSEA = 0.042, CFI = 0.963, SRMR = 0.029

### 4.6 Moderation analysis: role of digital learning experience

An exploratory moderation analysis was conducted using Hayes’ PROCESS macro (Model 1) to examine whether individual differences in digital learning experience influenced the relationship between PNI and attention allocation. Analyses used PROCESS Model 1 with heteroskedasticity-consistent SEs and 5,000 bootstrap resamples (Hayes, 2022). The digital learning experience was assessed through pre-study self-reported ratings of familiarity with online learning tools and the frequency of participation in virtual courses. The interaction between PNI and digital learning experience was statistically significant, *b* = 0.28 (unstandardized), SE = 0.11, *p* = 0.013, 95% CI [0.06, 0.50], indicating a small to moderate moderating effect. Learners with higher digital familiarity showed a less pronounced reduction in attention under high-distraction conditions compared to those with lower familiarity. However, no moderation effect emerged for cognitive load, suggesting that while prior digital experience may buffer against environmental interference, it does not appear to mitigate the cognitive strain caused by complex instructional design (Table 5).

**Table 5:** Moderation analysis: role of digital learning experience.

Moderator variable	Independent variable	Dependent variable	Interaction term (b, unstandardized)	Standard error (SE)	*p*-value	Interpretation
Digital learning experience	PNI	Attention Allocation	0.28	0.11	0.013	Higher digital familiarity reduces the negative impact of PNI on attention.

### 4.7 Exploratory behavioral indicators

Behavioral metrics provided additional support for the main findings. Participants in high PNI conditions exhibited significantly more task-switching events (*M* = 3.8, SD = 1.2) than those in low PNI conditions (*M* = 1.9, SD = 0.9), *t*(238) = 5.12, *p* < 0.001, Cohen’s *d* = 0.66, indicating a medium-to-large effect consistent with greater attentional fragmentation and reduced sustained focus. Similarly, response times to embedded comprehension prompts were significantly longer in high IDC conditions (*M* = 4.3 s, SD = 1.0) compared to low IDC conditions (*M* = 2.9 s, SD = 0.8), *t*(238) = 4.71, *p* < 0.001, *d* = 0.61, reflecting a medium effect size and suggesting increased cognitive processing demands under more complex instructional design. While these patterns align with the primary experimental results, they should be interpreted as supplementary indicators rather than definitive evidence of causal mechanisms, as they do not independently establish the direction of influence. The findings provide converging evidence that both PNI and IDC contribute to heightened cognitive strain and reduced learning efficiency (Table 6). The notification-related pattern is consistent with interruption/notification research, which shows costs even in the absence of user interaction (Ohly and Bastin, 2023). Additionally, first−/second-pass eye-movement indicators showed longer first-pass durations on AOI-Core under High IDC (vs. Low IDC), and greater second-pass time/regressions to AOI-Core when PNI was present, consistent with initial integration demands and subsequent repair/reintegration (Appendix Table A3).

**Table 6:** Exploratory behavioral indicators.

Condition comparison	Behavioral measure	Mean (high condition)	Mean (low condition)	*t*-value	df	*p*-value	Interpretation
High vs. Low PNI	Task-switching events	3.8	1.9	5.12	238	<0.001	Participants under high PNI switched tasks nearly twice as often.
High vs. Low IDC	Response time to comprehension prompts	4.3	2.9	4.71	238	<0.001	Participants with high IDC responded significantly more slowly, indicating greater cognitive effort.

### 4.8 Supplementary eye-tracking analyses

Event-locked analyses around pop-up onsets showed a bottom-up capture signature. With PNI present, TTFF to the pop-up was shortened and early (0–2000 ms), and the pop-up dwell increased. Both were modestly attenuated under low IDC; transition metrics quantified recovery. Pop-up → core transitions within 1,000 ms were higher when PNI was low and reduced when PNI was high, consistent with top-down suppression after exogenous onsets. For core AOIs, high IDC yielded a top-down integration/repair profile, longer first-pass gaze, and greater second-pass time/regressions, indicating increased goal-directed integration and later re-orientation. Full statistics are in Appendix Tables A1–A3.

## 5 Discussion

### 5.1 Key findings and hypotheses support

This study examined the interaction between IDC and PNI in influencing attention allocation and information retention in a virtual multimedia learning context, with attention serving as a mediating mechanism. All four hypotheses (H1–H4) were supported. Participants exposed to high IDC, implemented via redundant on-screen text, background music, and fragmented/split-attention layouts, allocated less attention to relevant content and showed lower retention than those in low-IDC conditions, confirming H1. This pattern is consistent with prior CLT evidence on split-attention and redundancy, as well as with reported costs of background music (see 3.1). Integrated (non-fragmented) layouts reliably outperform split-attention designs (*g* ≈ 0.63), and unnecessary redundancy impairs learning. Additionally, background music with lyrics tends to hinder memory and reading comprehension (Schroeder and Cenkci, 2018; Trypke et al., 2023; Souza and Barbosa, 2023).

Likewise, participants in high-PNI conditions (periodic task-irrelevant notifications) showed reduced attention allocation and lower retention than those in low-PNI settings, confirming H2. The pattern is consistent with a suppress-or-switch strategy to externally cued onsets (contingent capture and signal suppression), which nevertheless carries measurable costs (Remington et al., 2001; Gaspelin and Luck, 2018; Stothart et al., 2015). Also, this pattern aligns with the LC4MP’s resource-competition account of encoding (Fisher et al., 2018b; Rössler, 2017) and with meta-analytic evidence on media multitasking–related interference (Parry and Le Roux, 2021).

Moreover, the combination of high IDC and high PNI produced the lowest attention and retention (partial *η*^2^ ≈ 0.03 for both outcomes), confirming H3. As detailed in 4.3, the IDC × PNI interaction reflected attenuation rather than elimination of costs. Finally, bootstrapped SEM analyses indicated that attention allocation partially mediated the effects of both IDC (unstandardized indirect effect *b* = −2.14 percentage points, 95% CI [−3.21, −1.08]) and PNI (unstandardized indirect effect *b* = −1.78 percentage points, 95% CI [−2.85, −0.97]) on retention, confirming H4(a, b). Event-locked transition metrics (pop-up capture and recovery) and first−/second-pass indicators converged with the aggregate attention results, reinforcing that PNI primarily disrupts ongoing processing via transient capture/recovery dynamics, whereas IDC increases initial integration and later repair. This supports the view, corroborated by recent eye-tracking reviews in education, that visual attention can operate as a proximal mechanism linking conditions to learning outcomes (Deng and Gao, 2023).

### 5.2 Theoretical contributions

The confirmation of all four hypotheses adds conceptual clarity and empirical weight to three foundational cognitive frameworks, while also reframing the role of attention in digitally mediated learning. First, this study extends CLT by showing that design-induced demands (IDC) are influential and that their interaction with environmental interference (PNI) increases strain on limited capacity. Crucially, the mediation results indicate that attention is not a static prerequisite but instead a dynamic, continuously negotiated resource that channels the effects of instructional complexity and environmental noise into learning outcomes. Evidence from a meta-analysis of integrated vs. split-attention layouts and from a recent redundancy review is consistent with this interpretation (Schroeder and Cenkci, 2018; Trypke et al., 2023). This reconceptualization positions attention as an operational bottleneck that can be monitored and potentially regulated in real time within virtual environments, where competing demands are more pervasive than in face-to-face settings. By explicitly tying event-locked pop-up effects to bottom-up capture-and-pass core-AOI effects and linking these to top-down integration/repair, the study operationalizes attention as a dynamic control system that channels design features and environmental onsets into learning outcomes.

Second, the findings enrich LC4MP by showing dynamic allocation of attentional resources in response to message complexity and competing stimuli, with attention directly tied to encoding outcomes (Fisher et al., 2018a; Rössler, 2017). By combining real-time eye-tracking with behavioral indicators (time-on-task, response latency), the study captures micro-level fluctuations in attention allocation, as recommended by recent educational eye-tracking reviews (Deng and Gao, 2023). The results also align with evidence from media multitasking studies, which suggest that frequent switching undermines attentional control and integrative processing (Parry and Le Roux, 2021; Haverkamp et al., 2024). Meanwhile, the moderating effect of digital familiarity suggests that prior virtual learning experience can partially buffer these PNI-related effects. These contributions push theoretical boundaries by positioning attention as a measurable, adaptable, and more fragile cognitive mechanism in virtual learning contexts than in traditional ones.

### 5.3 Practical implications

From an applied perspective, this research offers actionable insights for instructional designers, educators, and educational technology developers. Given the validated effects of IDC, digital course materials should prioritize clarity, coherence, and simplicity. Overuse of redundant on-screen text or fragmented layouts should be minimized (Schroeder and Cenkci, 2018; Trypke et al., 2023), and music with lyrics should be avoided during learning because it tends to impair memory and reading performance (Souza and Barbosa, 2023; Cheah et al., 2022), particularly when learning goals are complex or unfamiliar. Given the PNI effects, minimize non-essential alerts during high-IDC segments and consider batching non-urgent notifications (Fitz et al., 2019). Because of high IDC and high PNI compound costs, avoid pairing complex materials with alert-rich environments, especially during assessment. Platforms can also offer lightweight attention-support features (e.g., brief refocus prompts, adaptive pacing) to reduce disengagement.

### 5.4 Limitations and future research directions

While the study advances understanding of how IDC and PNI interact, certain limitations suggest directions for further investigation. The laboratory-based design, although offering high control and ecological realism, limits generalizability; therefore, future work should test these effects in naturalistic contexts, such as MOOCs or blended classrooms. Attention was assessed using eye-tracking and behavioral engagement; however, integrating neurocognitive measures (e.g., EEG, fNIRS) could yield deeper insights into the mechanisms underlying attention shifts. The focus on Chinese undergraduates restricts cross-cultural applicability, highlighting the need to include diverse age groups and educational backgrounds. Moreover, other learner characteristics, such as cognitive style, intrinsic motivation, and multitasking habits, were not examined but may also substantially influence attention allocation.

We did not include a differentiated cognitive-load measure (intrinsic/extraneous/germane) in the main study. This limits construct-level inferences about whether IDC primarily raised perceived ECL and whether PNI secondarily elevated ECL. We opted against in-session CL ratings to avoid measurement reactivity and because single-item measures show mixed validity (Miles et al., 2020; French et al., 2021; Noetel et al., 2022). A concrete next step is to add validated multi-item I/E/G scales (Leppink et al., 2013; Klepsch et al., 2017) and a brief post-session appraisal of perceived externality/relevance (e.g., “*the pop-ups felt unrelated to the lesson*”; “*the extra on-screen text felt like required content*”), allowing tests of whether PNI elevates perceived ECL under IDC and whether learner appraisals mediate effects. Future work may orthogonally cross visual and auditory manipulations (IDC-Visual × IDC-Auditory × PNI Modality) with matched salience/onset to isolate channel-specific interactions.

Consequently, future research should build on these findings by testing targeted instructional manipulations, such as varying the timing and salience of distractions or using adaptive multimedia designs that adjust cognitive load in response to real-time attention data. Examining distinct learner profiles, including differences in working memory capacity, digital expertise, and multitasking tendencies, could clarify how personal traits interact with cognitive and environmental demands. Expanding the model to incorporate affective factors, such as frustration or fatigue, and utilizing longitudinal designs would help explain how prolonged exposure to high-load, high-distraction settings affects self-regulation and learning outcomes. Extending these approaches to mobile learning contexts where distractions are frequent would further enhance the practical relevance of this research in today’s increasingly digital education landscape.

## 6 Conclusion

This study demonstrated that IDC and PNI significantly impair learners’ attention allocation and information retention in virtual learning environments. Crucially, their joint presence produced compounded costs (partial η^2^ ≈ 0.03), indicating an interaction rather than merely additive influence, underscoring the cognitive strain imposed by complex instructional designs and environmental interference. Attention allocation partially mediated these effects, reinforcing its role as a central cognitive mechanism in digital learning. These findings have important implications for designing cognitively efficient, distraction-aware virtual education systems and highlight the need for learner training in attention management and multitasking.

## Data Availability

The original contributions presented in the study are included in the article/Supplementary material, further inquiries can be directed to the corresponding author.
