# Supplementary material for: Instructional design complexity and pop-up notification interference: effects on attention allocation and information retention in virtual classrooms
Source: Front Psychol. 2025 Nov 12;16:1618121. doi: 10.3389/fpsyg.2025.1618121 (PMC12646897; doi:10.3389/fpsyg.2025.1618121)
Supplement: Supplementary file 1 [file Appendix.docx]

**Instructional Design Complexity and Pop-Up Notification Interference: Effects on Attention Allocation and Information Retention in Virtual Classrooms**

**Appendix A. Eye-tracking variable definitions and analysis details**

- *TTFF (pop-up)*: latency from pop-up onset to first fixation on the pop-up AOI (ms).
- *Pop-up dwell (0–2000 ms)*: total fixation time on pop-up AOI in first 2 s post-onset.
- *Transition probability (pop-up → core)*: probability that the next fixation after a pop-up fixation lands on a core content AOI within 1000 ms. Core → pop-up computed analogously in a −2000–0 ms baseline.
- *First-pass gaze (core AOI)*: sum of fixations from first entry until first exit from the AOI.
- *Second-pass time (core AOI)*: sum of fixations upon re-entry to the same AOI after the first exit. Regression-back probability is the proportion of trials with any return to a previously viewed core AOI.
- *Models/transformations*: TTFF log-transformed; mixed-effects models with random intercepts for participants. 95% CIs reported for effects.

**Table A1. Event-locked pop-up metrics**

**A1a. Time-to-first-fixation to pop-up (ms)**

| **Condition** | **N (eye-tracking)** | **Mean (ms)** | **SE** | **95% CI (Lower)** | **95% CI (Upper)** |
| --- | --- | --- | --- | --- | --- |
| Low IDC + Low PNI | 58 | 540.0 | 15.76 | 509.10 | 570.90 |
| Low IDC + High PNI | 58 | 360.0 | 15.76 | 329.10 | 390.90 |
| High IDC + Low PNI | 58 | 560.0 | 15.76 | 529.10 | 590.90 |
| High IDC + High PNI | 58 | 380.0 | 15.76 | 349.10 | 410.90 |

*Notes : within-cell SD=120 ms; SE=SD/√58=15.76; CI=±1.96·SE.*

**A1b. Pop-up dwell (0–2000 ms window; ms)**

| **Condition** | **N (eye-tracking)** | **Mean (ms)** | **SE** | **95% CI (Lower)** | **95% CI (Upper)** |
| --- | --- | --- | --- | --- | --- |
| Low IDC + Low PNI | 58 | 330.0 | 21.01 | 288.82 | 371.18 |
| Low IDC + High PNI | 58 | 520.0 | 21.01 | 478.82 | 561.18 |
| High IDC + Low PNI | 58 | 310.0 | 21.01 | 268.82 | 351.18 |
| High IDC + High PNI | 58 | 480.0 | 21.01 | 438.82 | 521.18 |

*Notes : within-cell SD=160 ms; SE=160/√58=21.01; CI=±1.96·SE.*

**Table A2. Transition probabilities**

**A2a. Transition probability: Pop-up → Core AOI (within 1000 ms)**

| **Condition** | **N (eye-tracking)** | **Mean (p)** | **SE** | **95% CI (Lower)** | **95% CI (Upper)** |
| --- | --- | --- | --- | --- | --- |
| Low IDC + Low PNI | 58 | 0.620 | 0.016 | 0.589 | 0.651 |
| Low IDC + High PNI | 58 | 0.450 | 0.016 | 0.419 | 0.481 |
| High IDC + Low PNI | 58 | 0.580 | 0.016 | 0.549 | 0.611 |
| High IDC + High PNI | 58 | 0.400 | 0.016 | 0.369 | 0.431 |

**A2b. Transition probability: Core → Pop-up AOI (baseline; −2000–0 ms)**

| **Condition** | **N (eye-tracking)** | **Mean (p)** | **SE** | **95% CI (Lower)** | **95% CI (Upper)** |
| --- | --- | --- | --- | --- | --- |
| Low IDC + Low PNI | 58 | 0.060 | 0.016 | 0.029 | 0.091 |
| Low IDC + High PNI | 58 | 0.080 | 0.016 | 0.049 | 0.111 |
| High IDC + Low PNI | 58 | 0.050 | 0.016 | 0.019 | 0.081 |
| High IDC + High PNI | 58 | 0.090 | 0.016 | 0.059 | 0.121 |

*Notes : within-cell SD=0.12; SE=0.12/√58=0.01576; CI=±0.0309. Spatial AOI for “pop-up” is defined at its future location in the baseline to index anticipatory scanning.*

**Table A3. First-/Second-pass indicators on core AOIs**

**A3a. First-pass gaze duration (ms)**

| **Condition** | **N (eye-tracking)** | **Mean (ms)** | **SE** | **95% CI (Lower)** | **95% CI (Upper)** |
| --- | --- | --- | --- | --- | --- |
| Low IDC + Low PNI | 58 | 820.0 | 28.88 | 763.39 | 876.61 |
| Low IDC + High PNI | 58 | 760.0 | 28.88 | 703.39 | 816.61 |
| High IDC + Low PNI | 58 | 980.0 | 28.88 | 923.39 | 1036.61 |
| High IDC + High PNI | 58 | 910.0 | 28.88 | 853.39 | 966.61 |

*Notes : within-cell SD=220 ms; SE=220/√58=28.88; CI=±56.61.*

**A3b. Second-pass (rereading) time (ms)**

| **Condition** | **N (eye-tracking)** | **Mean (ms)** | **SE** | **95% CI (Lower)** | **95% CI (Upper)** |
| --- | --- | --- | --- | --- | --- |
| Low IDC + Low PNI | 58 | 240.0 | 23.64 | 193.66 | 286.34 |
| Low IDC + High PNI | 58 | 330.0 | 23.64 | 283.66 | 376.34 |
| High IDC + Low PNI | 58 | 360.0 | 23.64 | 313.66 | 406.34 |
| High IDC + High PNI | 58 | 470.0 | 23.64 | 423.66 | 516.34 |

*Notes : within-cell SD=180 ms; SE=180/√58=23.64; CI=±46.34.*

**A3c. Regression-back probability (return to a previously viewed core AOI)**

| **Condition** | **N (eye-tracking)** | **Mean (p)** | **SE** | **95% CI (Lower)** | **95% CI (Upper)** |
| --- | --- | --- | --- | --- | --- |
| Low IDC + Low PNI | 58 | 0.220 | 0.024 | 0.174 | 0.266 |
| Low IDC + High PNI | 58 | 0.300 | 0.024 | 0.254 | 0.346 |
| High IDC + Low PNI | 58 | 0.330 | 0.024 | 0.284 | 0.376 |
| High IDC + High PNI | 58 | 0.420 | 0.024 | 0.374 | 0.466 |

*Notes : within-cell SD=0.18; SE=0.18/√58=0.02364; CI=±0.04634.*


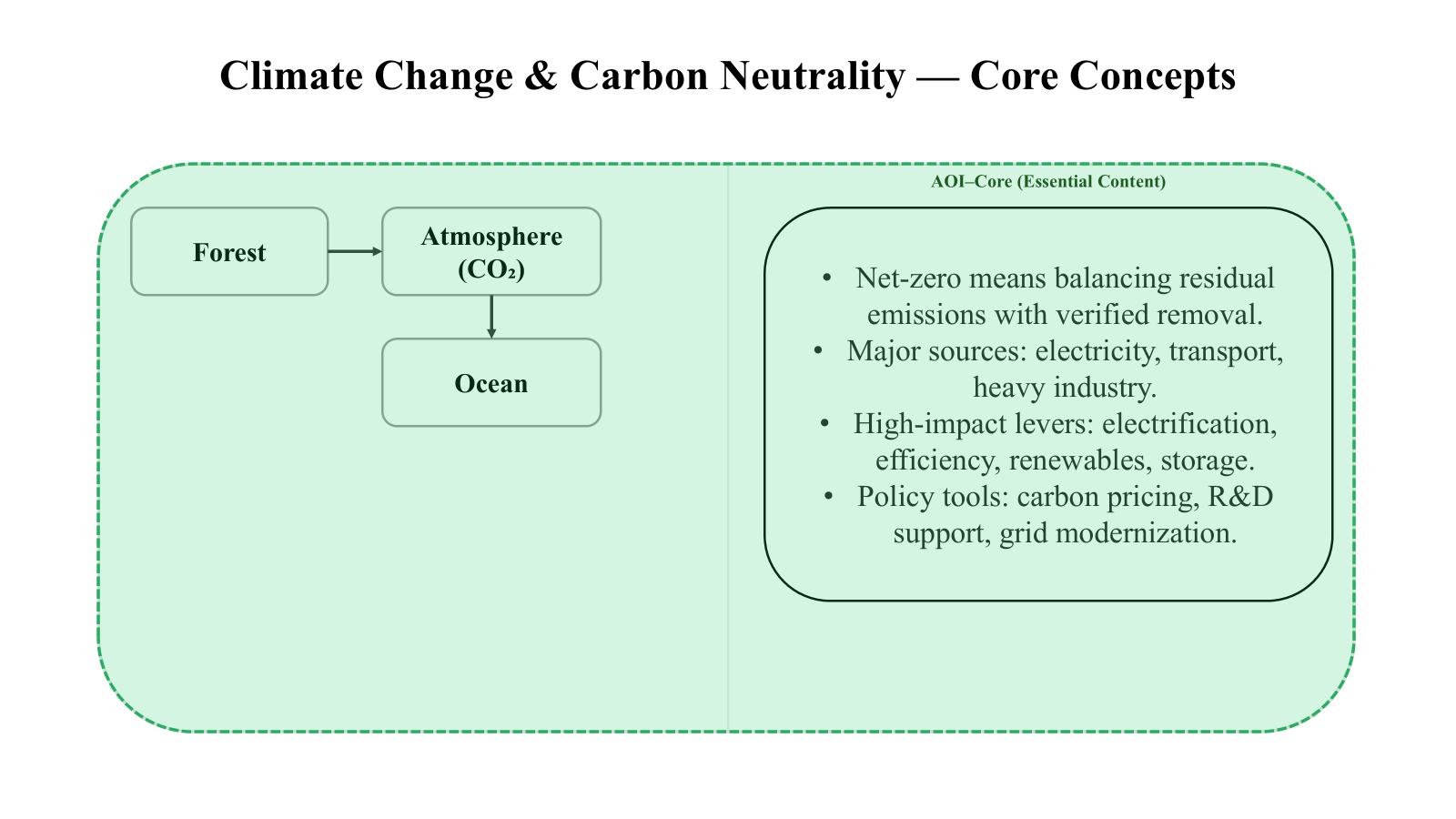


**Figure A1 — Low IDC + Low PNI**


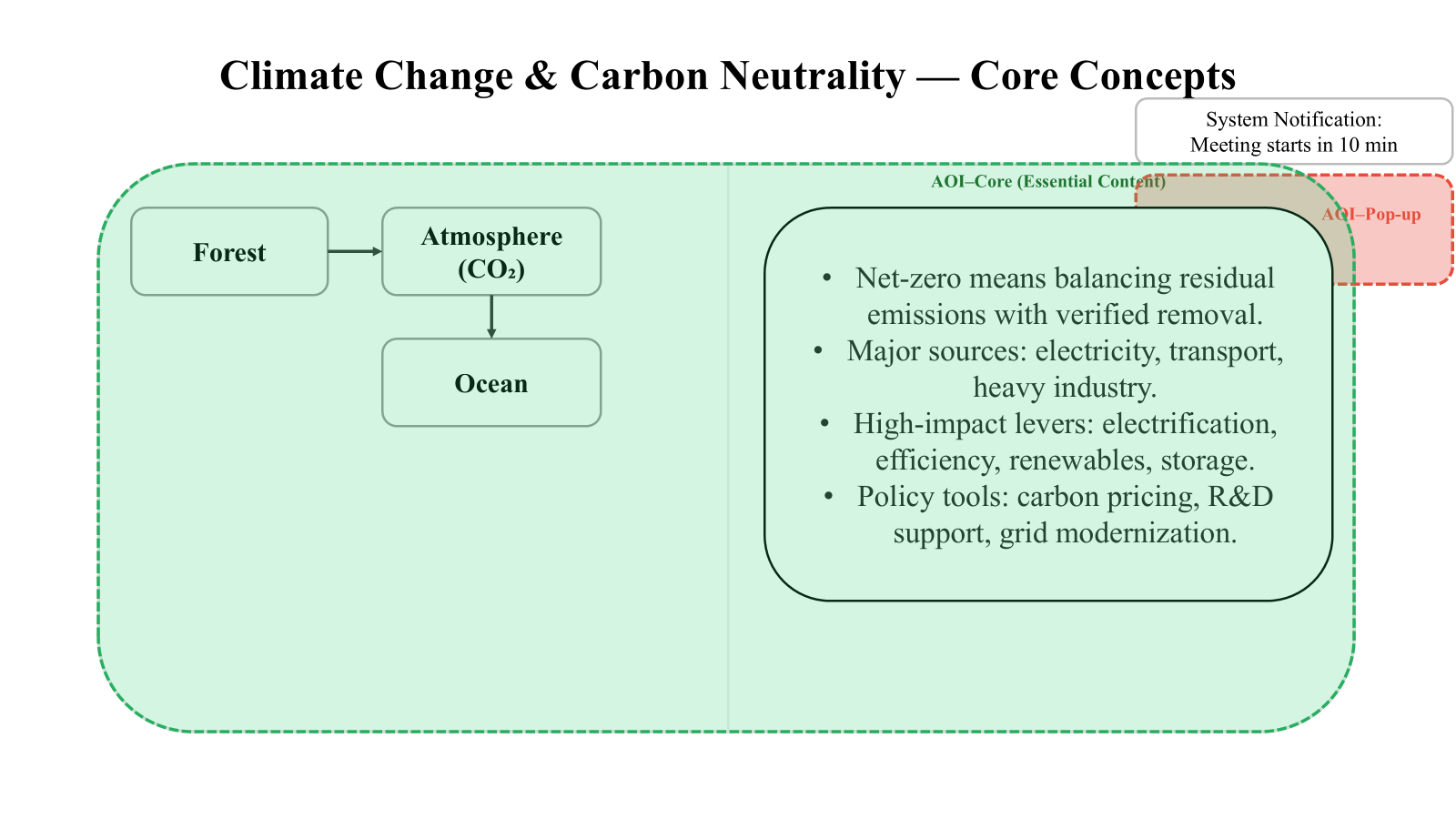


**Figure A2 — High IDC + Low PNI**


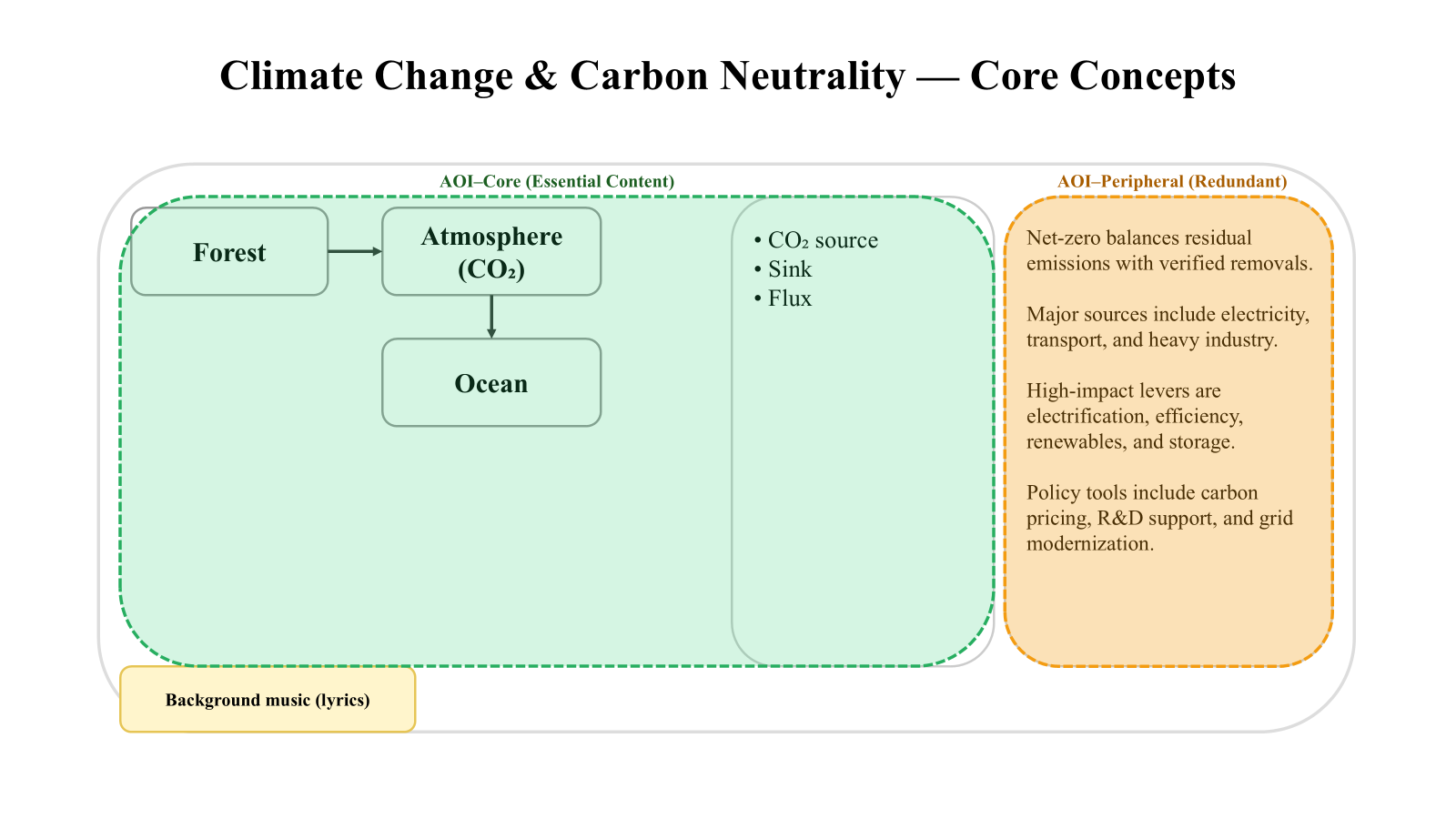


**Figure A3 — Low IDC + High PNI**


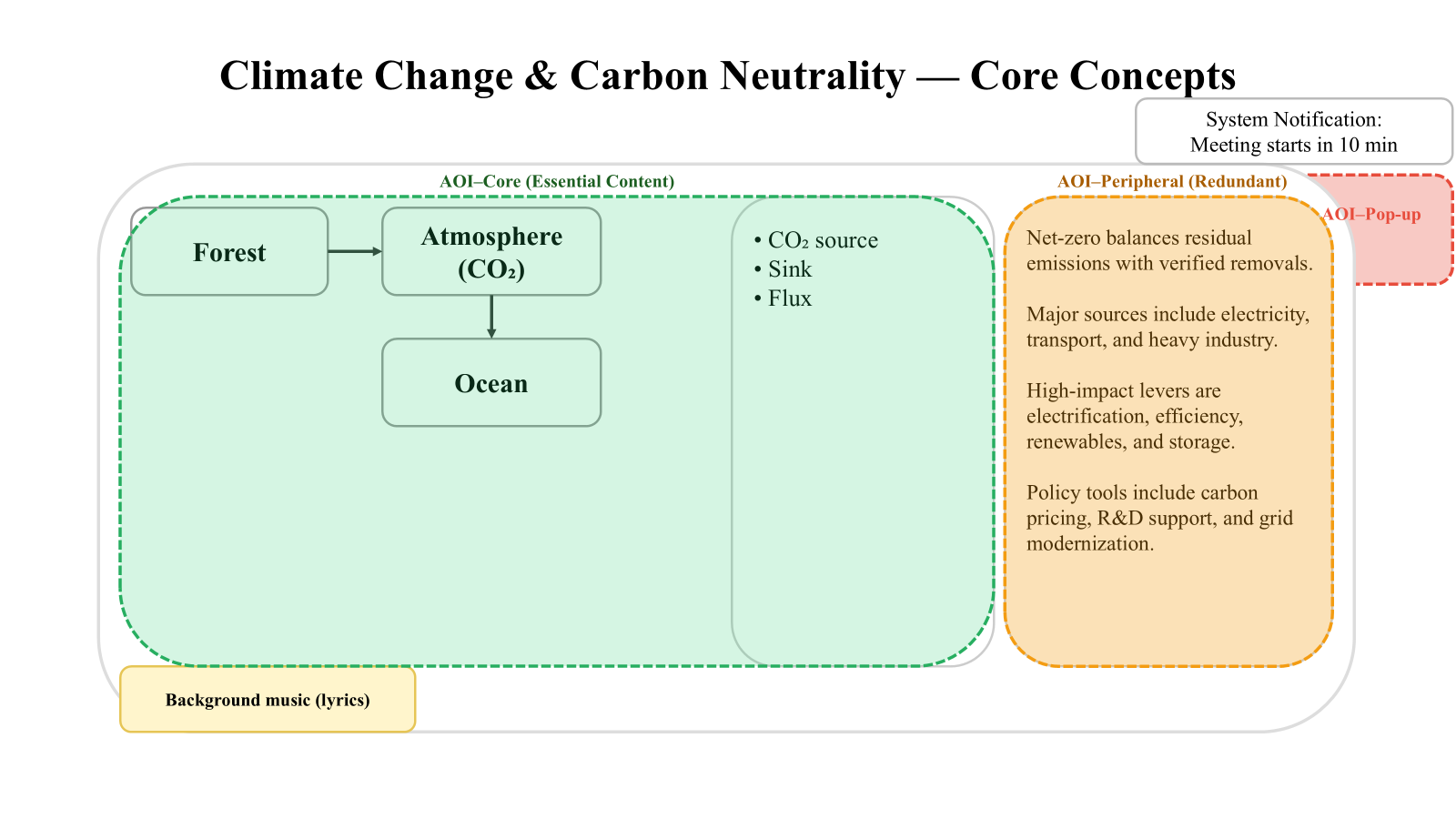


**Figure A4 — High IDC + High PNI**
